# Supplementary material for: Humanization of antibodies using a machine learning approach on large-scale repertoire data
Source: Bioinformatics. 2021 Jun 10;37(22):4041–7. doi: 10.1093/bioinformatics/btab434 (PMC8760955; doi:10.1093/bioinformatics/btab434)
Supplement: btab434_Supplementary_Data [file btab434_supplementary_data.zip › Supplementary Information - Main.docx]

**Supplementary Information: Humanization of antibodies using a machine learning approach on large-scale repertoire data**

Claire Marks^1^, Alissa M Hummer^1^, Mark Chin^1^ and Charlotte M Deane^1,^*

^1^ Department of Statistics, University of Oxford, Oxford, OX1 3LB, UK

* To whom correspondence should be addressed

Contents:

1. Data
   1. Overview of sequences downloaded from the Observed Antibody Space database
   2. Negative sequences – breakdown by species
   3. Therapeutics – sequences
   4. Therapeutics – origin species
   5. Therapeutics – ADA data
   6. Hu-mAb test set – references and reported immunogenicity

1. Methods
   1. ‘Infixes’ used for therapeutic classification
   2. Hu-mAb pipeline
   3. Residue type definitions for calculation of the Adjusted Overlap Ratio
2. Results
   1. PCA analysis of VH sequences by V gene and J gene type
   2. Classification performance of RF models
   3. Feature importance of RF models
   4. Comparison of our RF models with LSTM
   5. Classification of therapeutics using LSTM
   6. Relationship of RF humanness scores with immunogenicity
   7. Hu-mAb humanization results
   8. Random humanization results
   9. Negative control Hu-mAb humanization results
   10. Analysis of proposed mutations – residue types
   11. Analysis of proposed mutations – residue locations
3. **Data**
   1. Overview of sequences downloaded from the Observed Antibody Space database

Table S1. Numbers of human sequences downloaded from the Observed Antibody Space database after filtering.

|  | **VH** | **VL (kappa)** | **VL (lambda)** |
| --- | --- | --- | --- |
| V1 | 1,189,145 | 8,445,547 | 7,343,760 |
| V2 | 52,673 | 2,873,511 | 9,005,751 |
| V3 | 2,680,192 | 8,678,865 | 4,788,775 |
| V4 | 1,075,999 | 3,245,968 | 747,946 |
| V5 | 87,227 | 32,593 | 256,729 |
| V6 | 29,894 | 131,586 | 429,196 |
| V7 | 17,989 |  | 637,720 |
| V8 |  |  | 409,564 |
| V10 |  |  | 32,503 |
| **Total** | **5,133,119** | **23,408,070** | **23,209,877** |

Table S2. Numbers of negative (non-human) sequences downloaded from the Observed Antibody Space database after filtering.

|  | **VH** | **VL (kappa)** | **VL (lambda)** |
| --- | --- | --- | --- |
| **Total** | **12,284,297** | **950,335** | **655,826** |

- 1. Negative sequences - breakdown by species


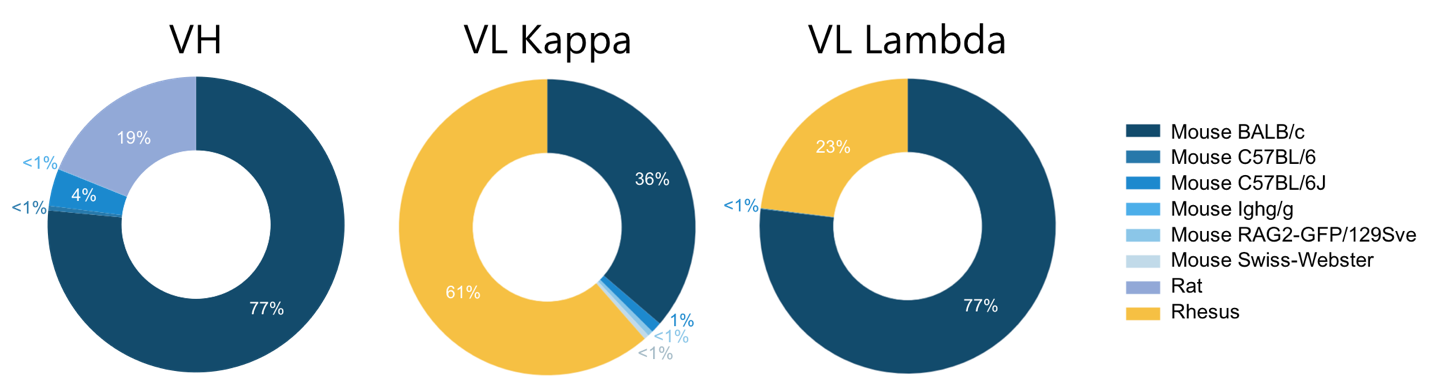


Figure S1. Breakdown by species of negative sequences downloaded from the Observed Antibody Space database after filtering.

- 1. Therapeutics – sequences

For the list of 481 therapeutics and their sequences, please see the SI file ‘Therapeutic_Sequences.xlsx’. The precursor and experimentally humanized sequences of the 25 therapeutics used to test Hu-mAb can be found in the SI file ‘Hu-mAb_Results.xlsx’.

- 1. Therapeutics - origin species


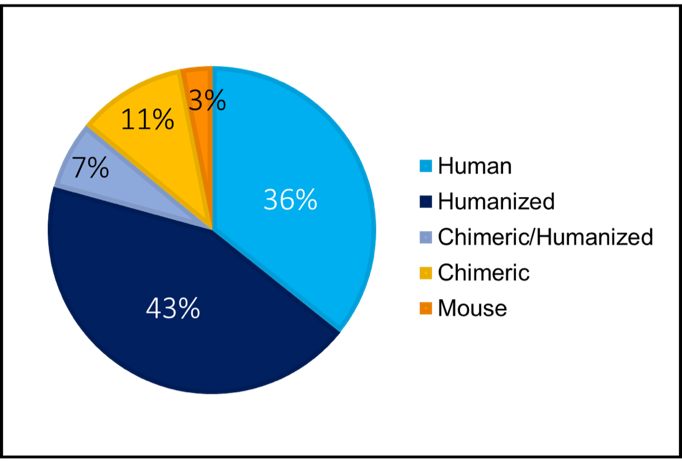


Figure S2. Therapeutic antibodies split by origin that are approved or in phase 1-3 trials. The therapeutics were gathered from the Therapeutic Structural Antibody Database (Raybould et al., 2020), which had a total of 481 antibody therapeutics intended for human use.

- 1. Therapeutics – ADA data

For the ADA data corresponding to therapeutics sequences, please see the SI file ‘Therapeutic_ADA.xlsx’.

- 1. Hu-mAb test set - references and reported immunogenicity

25 therapeutics were selected for which both the precursor and experimentally humanized sequences were available. The references for these sequences are as follows:

Table S3. References for precursor and experimentally humanized sequences of 25 therapeutics.

| **Therapeutic** | **Reference** |
| --- | --- |
| AntiCD28 | https://www.jimmunol.org/content/169/2/1119 |
| Campath | https://journals.lww.com/transplantjournal/Fulltext/1999/11150/ANTI_GLOBULIN_RESPONSES_TO_RAT_AND_HUMANIZED.32.aspx |
| Bevacizumab | http://www.imgt.org/IMGTrepertoire/GenesClinical/humanized/bevacizumab/bevacizumab_ProteinDisplay.html#igh |
| Herceptin | https://www.ncbi.nlm.nih.gov/pmc/articles/PMC49066/pdf/pnas01084-0075.pdf |
| Omalizumab | https://www.jimmunol.org/content/151/5/2623 |
| Eculizumab | https://patentimages.storage.googleapis.com/f2/c4/09/171125042450cd/EP2298808A1.pdf |
| Tocilizumab | https://cancerres.aacrjournals.org/content/canres/53/4/851.full.pdf |
| Pembrolizumab | https://cancerres.aacrjournals.org/content/74/19_Supplement/5024 |
| Pertuzumab | https://pubmed.ncbi.nlm.nih.gov/16151804/ |
| Ixekizumab | https://www.ncbi.nlm.nih.gov/pmc/articles/PMC4846058/#SD1-jir-9-039 |
| Palivizumab | https://academic.oup.com/jid/article/176/5/1215/831423 |
| Certolizumab | https://patentimages.storage.googleapis.com/54/71/03/400fe464c8bb2d/US20050042219A1.pdf |
| Idarucizumab | https://patentimages.storage.googleapis.com/51/ff/74/48026e9919861a/EP2525812B1.pdf |
| Reslizumab | https://patentimages.storage.googleapis.com/b1/be/fc/ee66a606c6ed4f/CA2192543C.pdf |
| Solanezumab | https://patentimages.storage.googleapis.com/8a/ed/d6/645d49a2b2fae4/WO2004071408A2.pdf |
| Lorvotuzumab | https://patentimages.storage.googleapis.com/6e/13/b2/f740eceb58298a/US5639641.pdf |
| Pinatuzumab | https://patentimages.storage.googleapis.com/e1/b4/6f/b94b77b6b5806f/ES2543475T3.pdf |
| Etaracizumab | https://www.pnas.org/content/pnas/95/15/8910.full.pdf |
| Talacotuzumab | https://patentimages.storage.googleapis.com/74/d1/05/9d3a61813b2985/US8492119.pdf |
| Rovalpituzumab | https://patentimages.storage.googleapis.com/4a/00/25/e01f76b1cb6ec6/US9089616.pdf |
| Clazakizumab | https://patentimages.storage.googleapis.com/52/b8/4f/0146181ade3705/US20090104187A1.pdf |
| Ligelizumab | https://patentimages.storage.googleapis.com/9d/5c/b5/f8789f9a5c7722/US7531169.pdf |
| Crizanlizumab | https://patentimages.storage.googleapis.com/b9/22/2d/5e02e51d7e935a/US8377440.pdf |
| Mogamulizumab | https://patentimages.storage.googleapis.com/20/fc/8e/b206ab42434698/US8491902.pdf |
| Refanezumab | https://patentimages.storage.googleapis.com/1b/f7/0c/0e94c7d7cf18ad/US8974782.pdf |

Reported immunogenicity for these 25 therapeutics is shown in the table below. All therapeutics except for two (Talacotuzumab, AntiCD28) have low immunogenicity (<10% patients with ADA). Immunogenicity of Anti-CD28 was measured in vitro using a mixed lymphocyte reaction (MLR) assay and the precursor sequence was found to have a much higher immunogenic response compared to the experimental humanized sequence.

Table S4. Reported immunogenicity of 25 therapeutics with publicly available precursor and experimentally humanized sequences

| **Therapeutic** | **Immunogenicity (% of patients with ADA)** |
| --- | --- |
| AntiCD28 | NA |
| Campath | 5.10 |
| Bevacizumab | 0.32 |
| Herceptin | 8.10 |
| Omalizumab | 0.00 |
| Eculizumab | 2.00 |
| Tocilizumab | 2.00 |
| Pembrolizumab | 1.70 |
| Pertuzumab | 2.80 |
| Ixekizumab | 8.50 |
| Palivizumab | 1.10 |
| Certolizumab | 8.00 |
| Idarucizumab | 4.00 |
| Reslizumab | 5.00 |
| Solanezumab | 3.50 |
| Lorvotuzumab | 0.00 |
| Pinatuzumab | 1.40 |
| Etaracizumab | 0.00 |
| Talacotuzumab | 17.40 |
| Rovalpituzumab | 0.00 |
| Clazakizumab | 1.82 |
| Ligelizumab | 6.32 |
| Crizanlizumab | 0.94 |
| Mogamulizumab | 4.20 |
| Refanezumab | 9.38 |

1. Methods
   1. ‘Infixes’ used for therapeutic classification

Table S5. ‘Infixes’ used for therapeutic classification

| **Source infix** | **Origin** |
| --- | --- |
| -u- | Human |
| -zu- | Humanized |
| -xizu- | Chimeric/Humanized |
| -xi- | Chimeric |
| -o- | Mouse |

- 1. Hu-mAb pipeline

Figure S3. Outline of the Hu-mAb protocol. Input sequences are scored by RF models; if the humanness threshold is not reached, the model with the highest score is selected for humanization. All possible mutations within the framework region of the input sequence are made and each sequence scored by the respective RF model. The mutated sequence with the highest humanness score is selected and the process repeated until the target humanness threshold is achieved.

- 1. Residue type definitions for calculation of the Adjusted Overlap Ratio

Groupings of amino acid types used to calculate the Adjusted Overlap Ratio (AOR). The AOR treats any mutation of the same type as an overlapping / identical mutation. For example, if the experimental mutation was R to A and the model mutation was R to V, this would still be considered an overlap. Others are not included as part of the AOR calculation and each AA is treated as a separate type.

Table S6. Amino acid groupings based on physicochemical characteristics. Amino acids are denoted in single-letter code.

| **Type** | **Amino Acids** |
| --- | --- |
| Positive | K, R, H |
| Negative | D, E |
| Hydrophobic | V, M, I, L, A |
| Hydrophilic | Q, N, S, T |
| Aromatic | W, F, Y |
| Others | C, G, P |

1. Results
   1. PCA analysis of VH sequences by V gene and J gene type

The variable domain of each antibody is predominantly made from a single V gene and we expected that sequences from the same V gene would be more similar than sequences from other V genes. Moreover, we envisaged the humanizer to propose mutations towards a particular V gene type rather than an unrealistic hybrid sequence. We investigated the dissimilarity across sequences from different gene types via Principal Component Analysis (PCA). All PCA analysis was carried out using the scikit-learn Python PCA module. Input sequences were one-hot encoded, and the resulting vectors standardized using the scikit-learn Python StandardScaler module, for the PCA.

All human IgG VH sequences were downloaded from OAS (Kovaltsuk et al., 2018) and prepared as described in the Methods. Each sequence was labelled by its V gene type (V1-V7). The PCA results demonstrated that each set of V gene sequences formed clusters that were completely separable from all other sets of V gene sequences with just two or three principal components (Figure S4A-D).

The same approach was performed with J genes. Each set of V gene sequences were split into their respective J gene types (J1-J6). PCA was performed on each set of V gene sequences and the separated sequences were labelled by their J gene type. Figure S4E shows no distinct clustering across the V3 J gene sequences, which were completely inseparable across all three principal components. The lack of separability by J gene type was consistent across all sets of V gene sequences. Therefore, we only split sequences by V gene and not J gene type for RF model construction.


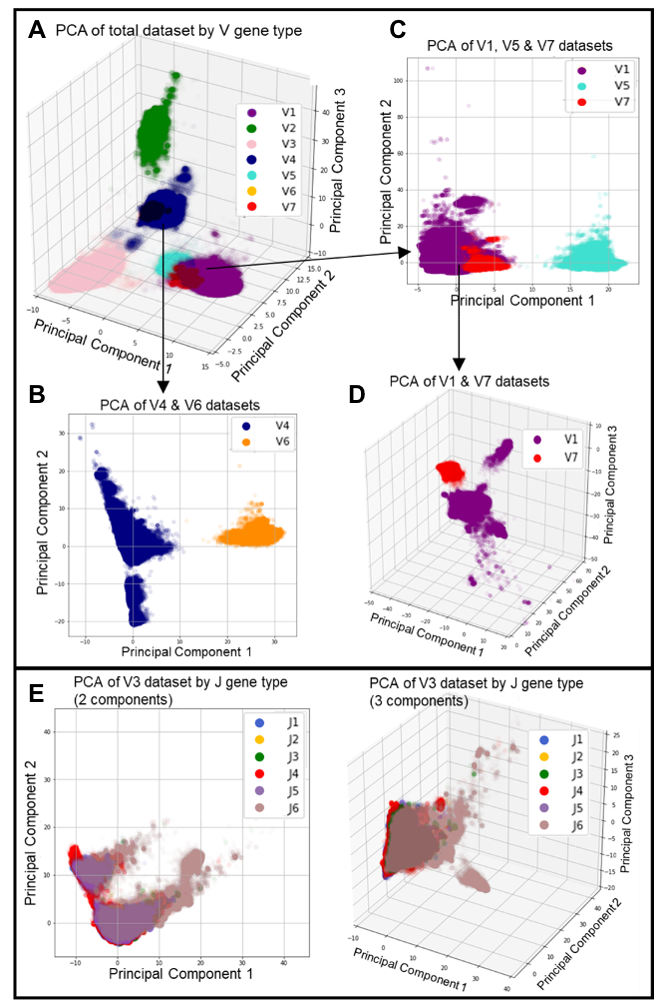


Figure S4. Principal Component Analysis of VH sequences by V gene type and J gene type. Sequences were labelled by their V gene type (A,B,C,D) or by their J gene type (E). A) Three component PCA of all VH sequences exhibited four distinct clusters of sequences; consisting of (i) V2, (ii) V3, (iii) V4/V6 and (iv) V1/V5/V7. Further PCA was performed independently on sequences in cluster (iii) and (iv). B) Two component PCA of V4 and V6 sequences exhibited distinct clustering of each set. C) Two component PCA of V1, V5 and V7 sequences resulted in two distinct clusters; V1/V7 and V5. Further PCA was performed on just V1 and V7. D) Three component PCA of V1 and V7 sequences exhibited distinct clustering of each set. E) Two component and three component PCA of V3 sequences labelled by their J gene type. No distinct clustering was observed with all sets of J gene sequences overlapped.

- 1. Classification performance of RF models

Validation and Test Set performance for each RF model. Extremely high performance was observed across all models demonstrating AUC scores close to 1. The VH models were able to perfectly discriminate between human and non-human sequences with AUC scores of 1. YJS scores were similar in both validation and test sets with all models scoring ≥0.999. MCC thresholds obtained using the validation set were very similar to the YJS thresholds – the YJS thresholds were taken forward for use in subsequent experiments.

Table S7. Validation performance of RF models.

|  | **V Gene** | **ROCAUC** | **YJS** | **YJS Threshold** | **MCC** | **MCC Threshold** |
| --- | --- | --- | --- | --- | --- | --- |
| H  E  A  V  Y | HV1 | 1.0000000000 | 1.000000 | 0.565 | 1.000000 | 0.565 |
|  | HV2 | 1.0000000000 | 1.000000 | 0.615 | 1.000000 | 0.615 |
|  | HV3 | 1.0000000000 | 1.000000 | 0.630 | 1.000000 | 0.630 |
|  | HV4 | 1.0000000000 | 1.000000 | 0.495 | 1.000000 | 0.495 |
|  | HV5 | 1.0000000000 | 1.000000 | 0.480 | 1.000000 | 0.480 |
|  | HV6 | 1.0000000000 | 1.000000 | 0.640 | 1.000000 | 0.640 |
|  | HV7 | 1.0000000000 | 1.000000 | 0.575 | 1.000000 | 0.575 |
| K  A  P  P  A | KV1 | 0.9999990912 | 0.999906 | 0.740 | 0.999538 | 0.714 |
|  | KV2 | 0.9999999990 | 0.999986 | 0.602 | 0.999972 | 0.642 |
|  | KV3 | 0.9999999989 | 0.999977 | 0.845 | 0.999737 | 0.722 |
|  | KV4 | 0.9999999998 | 0.999994 | 0.650 | 0.999993 | 0.650 |
|  | KV5 | 1.0000000000 | 1.000000 | 0.515 | 1.000000 | 0.515 |
|  | KV6 | 1.0000000000 | 1.000000 | 0.490 | 1.000000 | 0.490 |
| L  A  M  B  D  A | LV1 | 0.9999999999 | 0.999992 | 0.856 | 0.999988 | 0.816 |
|  | LV2 | 0.9999999996 | 0.999997 | 0.790 | 0.999984 | 0.772 |
|  | LV3 | 0.9999999990 | 0.999967 | 0.770 | 0.999965 | 0.800 |
|  | LV4 | 0.9999999990 | 0.999973 | 0.815 | 0.999986 | 0.800 |
|  | LV5 | 0.9999999994 | 0.999985 | 0.370 | 0.999973 | 0.510 |
|  | LV6 | 1.0000000000 | 1.000000 | 0.800 | 1.000000 | 0.800 |
|  | LV7 | 1.0000000000 | 1.000000 | 0.810 | 1.000000 | 0.810 |
|  | LV8 | 0.9999999985 | 0.999985 | 0.754 | 0.999990 | 0.754 |
|  | LV10 | 1.0000000000 | 1.000000 | 0.625 | 1.000000 | 0.590 |

Table S8. Testing performance of RF models.

|  | **V Gene** | **ROCAUC** | **YJS** | **MCC** |
| --- | --- | --- | --- | --- |
| H  E  A  V  Y | HV1 | 1.00000000000 | 1.000000 | 1.000000 |
|  | HV2 | 1.00000000000 | 1.000000 | 1.000000 |
|  | HV3 | 1.00000000000 | 1.000000 | 1.000000 |
|  | HV4 | 1.00000000000 | 1.000000 | 1.000000 |
|  | HV5 | 1.00000000000 | 1.000000 | 1.000000 |
|  | HV6 | 1.00000000000 | 1.000000 | 1.000000 |
|  | HV7 | 1.00000000000 | 1.000000 | 1.000000 |
| K  A  P  P  A | KV1 | 0.99999981855 | 0.999552 | 0.999540 |
|  | KV2 | 0.99999999844 | 0.999958 | 0.999970 |
|  | KV3 | 0.99999999770 | 0.999526 | 0.999740 |
|  | KV4 | 0.99999999998 | 0.999997 | 0.999990 |
|  | KV5 | 1.00000000000 | 1.000000 | 1.000000 |
|  | KV6 | 1.00000000000 | 1.000000 | 1.000000 |
| L  A  M  B  D  A | LV1 | 0.99999999994 | 0.999996 | 0.999980 |
|  | LV2 | 0.99999999998 | 0.999997 | 0.999980 |
|  | LV3 | 0.99999998860 | 0.999950 | 0.999960 |
|  | LV4 | 1.00000000000 | 0.999987 | 0.999990 |
|  | LV5 | 0.99999999941 | 0.999995 | 0.999920 |
|  | LV6 | 1.00000000000 | 1.000000 | 1.000000 |
|  | LV7 | 1.00000000000 | 1.000000 | 1.000000 |
|  | LV8 | 1.00000000000 | 1.000000 | 1.000000 |
|  | LV10 | 1.00000000000 | 0.999692 | 0.999840 |

Table S9. Testing performance of RF models on a subset of the test dataset limited to sequences with <97% sequence identity with any training/validation sequence.

|  | **V Gene** | **ROCAUC** | **YJS** | **MCC** |
| --- | --- | --- | --- | --- |
| H  E  A  V  Y | HV1 | 1.00000000000 | 1.000000 | 1.000000 |
|  | HV2 | 1.00000000000 | 1.000000 | 1.000000 |
|  | HV3 | 1.00000000000 | 1.000000 | 1.000000 |
|  | HV4 | 1.00000000000 | 1.000000 | 1.000000 |
|  | HV5 | 1.00000000000 | 1.000000 | 1.000000 |
|  | HV6 | 1.00000000000 | 1.000000 | 1.000000 |
|  | HV7 | 1.00000000000 | 1.000000 | 1.000000 |
| K  A  P  P  A | KV1 | 0.99999910846 | 0.999526 | 0.999344 |
|  | KV2 | 0.99999998828 | 0.999918 | 0.999937 |
|  | KV3 | 0.99999999130 | 0.999894 | 0.999760 |
|  | KV4 | 1.00000000000 | 0.999990 | 0.999985 |
|  | KV5 | 1.00000000000 | 1.000000 | 1.000000 |
|  | KV6 | 1.00000000000 | 1.000000 | 1.000000 |
| L  A  M  B  D  A | LV1 | 0.99999999897 | 0.999978 | 0.999820 |
|  | LV2 | 0.99999999982 | 0.999997 | 0.999970 |
|  | LV3 | 0.99999996100 | 0.999818 | 0.999844 |
|  | LV4 | 1.00000000000 | 0.999965 | 0.999954 |
|  | LV5 | 0.99999999418 | 0.999831 | 0.999761 |
|  | LV6 | 1.00000000000 | 1.000000 | 1.000000 |
|  | LV7 | 1.00000000000 | 1.000000 | 1.000000 |
|  | LV8 | 1.00000000000 | 1.000000 | 1.000000 |
|  | LV10 | 1.00000000000 | 0.999217 | 0.999580 |

- 1. Feature importance of RF models

Figure S5. Feature importance of heavy chain RF models. The x-axis consists of the residue positions in a sequential manner (left to right, IMGT numbering scheme). The most important features likely determine the humanness of the sequence.

Figure S6. Feature importance of kappa light chain RF models. The x-axis consists of the residue positions in a sequential manner (left to right, IMGT numbering scheme). The most important features likely determine the humanness of the sequence.

Figure S7. Feature importance of lambda light chain RF models. The x-axis consists of the residue positions in a sequential manner (left to right, IMGT numbering scheme). The most important features likely determine the humanness of the sequence.

- 1. Comparison of our RF models with LSTM

Our RF models outperform the LSTM equivalents in both AUC and YJS scores for each model.

Table S10. Comparison of our RF models with LSTM models in performance

|  |  | **Random Forest** | | **LSTM** | |
| --- | --- | --- | --- | --- | --- |
|  | **V Gene** | **ROCAUC** | **YJS** | **ROCAUC** | **YJS** |
| H  E  A  V  Y | HV1 | 1.0000000000 | 1.000000 | 0.999772 | 0.9960 |
|  | HV2 | 1.0000000000 | 1.000000 | 0.999996 | 0.9970 |
|  | HV3 | 1.0000000000 | 1.000000 | 0.994383 | 0.9418 |
|  | HV4 | 1.0000000000 | 1.000000 | 0.991764 | 0.9917 |
|  | HV5 | 1.0000000000 | 1.000000 | 0.999954 | 0.9981 |
|  | HV6 | 1.0000000000 | 1.000000 | 0.999999 | 0.9997 |
|  | HV7 | 1.0000000000 | 1.000000 | 0.999991 | 0.9991 |
| K  A  P  P  A | KV1 | 0.9999998186 | 0.999552 | 0.939153 | 0.6790 |
|  | KV2 | 0.9999999984 | 0.999958 | 0.997548 | 0.9481 |
|  | KV3 | 0.9999999977 | 0.999526 | 0.993947 | 0.9156 |
|  | KV4 | 1.0000000000 | 0.999997 | 0.998431 | 0.9746 |
|  | KV5 | 1.0000000000 | 1.000000 | 0.999992 | 0.9993 |
|  | KV6 | 1.0000000000 | 1.000000 | 0.999683 | 0.9930 |
| L  A  M  B  D  A | LV1 | 0.9999999999 | 0.999996 | 0.998347 | 0.9702 |
|  | LV2 | 1.0000000000 | 0.999997 | 0.995076 | 0.9191 |
|  | LV3 | 0.9999999886 | 0.999950 | 0.999284 | 0.9740 |
|  | LV4 | 1.0000000000 | 0.999987 | 0.999989 | 0.9989 |
|  | LV5 | 0.9999999994 | 0.999995 | 0.999981 | 0.9959 |
|  | LV6 | 1.0000000000 | 1.000000 | 0.999962 | 0.9939 |
|  | LV7 | 1.0000000000 | 1.000000 | 0.999802 | 0.9919 |
|  | LV8 | 1.0000000000 | 1.000000 | 0.999999 | 0.9996 |
|  | LV10 | 1.0000000000 | 0.999692 | 0.999732 | 0.9933 |

- 1. Classification of therapeutics using LSTM


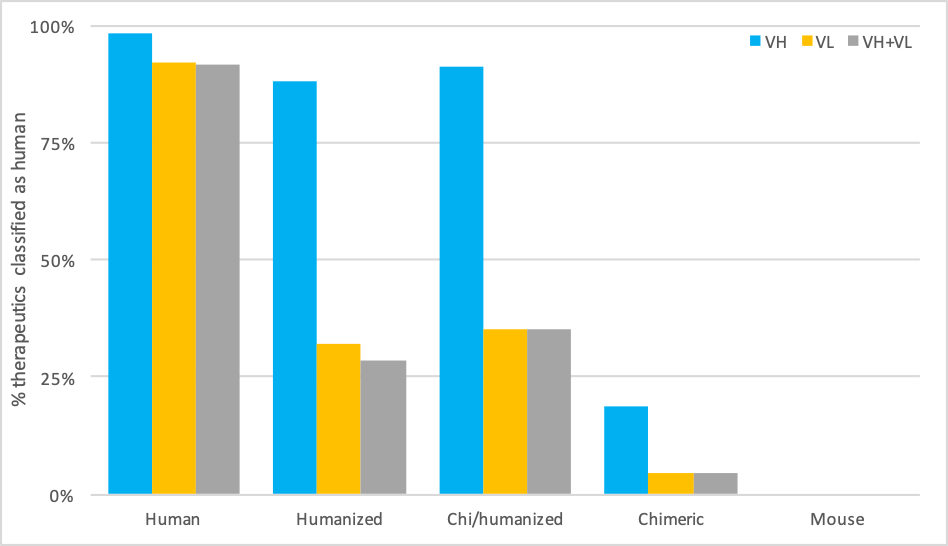


Figure S8. Percentage of antibody therapeutics classified as human by the LSTM method, split by their origin. Chi/humanized are sequences which are part humanized and part chimeric. Therapeutics were classified based on their VH and VL sequences separately, as well as combined (to be classified as human, both VH and VL scores had to be above the respective humanness threshold). For our RF models, the predicted humanness of the therapeutics decreased as the human content of the sequence decreases (left to right in the figure). This trend is also observed using the LSTM method, but not as clearly - for example, more of the chi/humanized set are classified as human than the humanized set, and more human therapeutics are classified as non-human.

- 1. Relationship of RF humanness scores with immunogenicity

Figure S9. Scatter plot comparing experimental immunogenicity (fraction of patients that develop ADAs) and the RF humanness score of therapeutic mAbs. A) VH sequences (Pearson correlation: r = -0.58, p = 5.59x10^-21^). B) VL sequences (Pearson correlation: r = -0.56, p = 4.29x10^-19^). C) VH/VL – the minimum humanness score of the respective VH and VL sequences (Pearson correlation: r = -0.56, p = 2.63x10^-19^). The Pearson correlation coefficient was calculated using the scipy.stats.pearsonr Python module.

- 1. Hu-mAb humanization results

For full results, please see SI file ‘Hu-mAb_Results.xlsx’. This file contains the precursor, experimentally humanized and Hu-mAb output sequences for each of the 25 therapeutics.

Table S11. Summary of humanization results for heavy chain sequences.

| **Therapeutic** | **V Gene** | **Initial Score** | **Target Score** | **# Exp. Mutations** | **# Hu-mAb Mutations** | **Mutation Ratio** | **Overlap Ratio** | **Adjusted OR** |
| --- | --- | --- | --- | --- | --- | --- | --- | --- |
| AntiCD28 | hv3 | 0.005 | 0.990 | 33 | 19 | 58% | 63% | 79% |
| Campath | hv4 | 0.000 | 0.835 | 39 | 16 | 41% | 75% | 88% |
| Bevacizumab | hv3 | 0.050 | 0.805 | 25 | 14 | 56% | 50% | 57% |
| Herceptin | hv3 | 0.000 | 0.965 | 32 | 27 | 84% | 59% | 78% |
| Omalizumab | hv3 | 0.000 | 0.955 | 34 | 21 | 62% | 62% | 76% |
| Eculizumab | hv1 | 0.000 | 0.975 | 23 | 15 | 65% | 73% | 73% |
| Tocilizumab | hv4 | 0.000 | 0.935 | 23 | 14 | 61% | 64% | 86% |
| Pembrolizumab | hv1 | 0.000 | 0.880 | 23 | 11 | 48% | 73% | 73% |
| Pertuzumab | hv3 | 0.000 | 0.875 | 32 | 19 | 59% | 68% | 79% |
| Ixekizumab | hv1 | 0.000 | 0.900 | 29 | 12 | 41% | 75% | 75% |
| Palivizumab | hv2 | 0.000 | 0.885 | 18 | 12 | 67% | 75% | 83% |
| Certolizumab | hv3 | 0.000 | 0.855 | 31 | 18 | 58% | 61% | 78% |
| Idarucizumab | hv4 | 0.000 | 0.760 | 24 | 15 | 63% | 80% | 80% |
| Reslizumab | hv3 | 0.060 | 0.760 | 21 | 10 | 48% | 50% | 80% |
| Solanezumab | hv3 | 0.005 | 0.855 | 16 | 10 | 63% | 50% | 70% |
| Lorvotuzumab | hv3 | 0.010 | 0.985 | 13 | 10 | 77% | 90% | 90% |
| Pinatuzumab | hv3 | 0.000 | 0.860 | 33 | 23 | 70% | 61% | 78% |
| Etaracizumab | hv3 | 0.005 | 0.940 | 16 | 12 | 75% | 58% | 83% |
| Talacotuzumab | hv5 | 0.000 | 0.815 | 33 | 18 | 55% | 78% | 83% |
| Rovalpituzumab | hv1 | 0.000 | 0.975 | 30 | 21 | 70% | 67% | 67% |
| Clazakizumab | hv3 | 0.645 | 0.995 | 27 | 7 | 26% | 86% | 86% |
| Ligelizumab | hv1 | 0.000 | 0.805 | 21 | 11 | 52% | 64% | 64% |
| Crizanlizumab | hv1 | 0.000 | 0.865 | 23 | 11 | 48% | 64% | 64% |
| Mogamulizumab | hv3 | 0.050 | 0.760 | 15 | 6 | 40% | 67% | 67% |
| Refanezumab | hv7 | 0.025 | 0.860 | 17 | 15 | 88% | 87% | 87% |
| **MEAN** |  |  |  |  |  | **59%** | **68%** | **77%** |
| **MEDIAN** |  |  |  |  |  | **59%** | **67%** | **78%** |

Table S12. Summary of humanization results for light chain sequences.

| **Therapeutic** | **V Gene** | **Initial Score** | **Target Score** | **# Exp. Mutations** | **# Hu-mAb Mutations** | **Mutation Ratio** | **Overlap Ratio** | **Adjusted OR** |
| --- | --- | --- | --- | --- | --- | --- | --- | --- |
| AntiCD28 | kv4 | 0.050 | 0.805 | 19 | 11 | 58% | 64% | 73% |
| Campath | kv1 | 0.724 | 0.842 | 14 | 3 | 21% | 67% | 67% |
| Bevacizumab | kv1 | 0.017 | 0.899 | 16 | 9 | 56% | 89% | 100% |
| Herceptin | kv1 | 0.032 | 0.776 | 22 | 8 | 36% | 88% | 88% |
| Omalizumab | kv1 | 0.081 | 0.874 | 25 | 19 | 76% | 89% | 95% |
| Eculizumab | kv1 | 0.002 | 0.893 | 20 | 12 | 60% | 83% | 83% |
| Tocilizumab | kv1 | 0.001 | 0.650 | 19 | 9 | 47% | 78% | 89% |
| Pembrolizumab | kv3 | 0.010 | 0.870 | 20 | 12 | 60% | 75% | 75% |
| Pertuzumab | kv1 | 0.006 | 0.888 | 20 | 10 | 50% | 80% | 90% |
| Ixekizumab | kv2 | 0.000 | 0.864 | 12 | 9 | 75% | 78% | 100% |
| Palivizumab | kv1 | 0.199 | 0.876 | 26 | 13 | 50% | 77% | 92% |
| Certolizumab | kv1 | 0.011 | 0.862 | 20 | 10 | 50% | 80% | 90% |
| Idarucizumab | kv2 | 0.258 | 0.900 | 8 | 6 | 75% | 67% | 67% |
| Reslizumab | kv1 | 0.389 | 0.791 | 20 | 6 | 30% | 83% | 100% |
| Solanezumab | kv2 | 0.060 | 0.888 | 10 | 8 | 80% | 88% | 100% |
| Lorvotuzumab | kv2 | 0.050 | 0.924 | 13 | 11 | 85% | 82% | 82% |
| Pinatuzumab | kv1 | 0.002 | 0.741 | 23 | 19 | 83% | 74% | 79% |
| Etaracizumab | kv3 | 0.010 | 0.950 | 25 | 13 | 52% | 62% | 69% |
| Talacotuzumab | kv4 | 0.005 | 0.935 | 16 | 11 | 69% | 73% | 73% |
| Rovalpituzumab | kv3 | 0.000 | 0.980 | 26 | 14 | 54% | 64% | 79% |
| Clazakizumab | kv1 | 0.778 | 0.911 | 22 | 4 | 18% | 75% | 75% |
| Ligelizumab | kv3 | 0.020 | 0.930 | 21 | 11 | 52% | 64% | 91% |
| Crizanlizumab | kv1 | 0.001 | 0.875 | 23 | 20 | 87% | 85% | 95% |
| Mogamulizumab | kv2 | 0.036 | 0.792 | 12 | 6 | 50% | 67% | 67% |
| Refanezumab | kv4 | 0.000 | 1.000 | 17 | 12 | 71% | 92% | 100% |
| **MEAN** |  |  |  |  |  | **58%** | **77%** | **85%** |
| **MEDIAN** |  |  |  |  |  | **56%** | **78%** | **88%** |

- 1. Random humanization results

Random humanization of selected therapeutics. A random humanization model was constructed to generate mutations randomly up to the same number of mutations as Hu-mAb. 100 million randomly humanized VH sequences were generated and the average Overlap Ratios and Adjusted Overlap Ratios were calculated.

Table S13. Random humanization of Certolizumab, Omalizumab, Eculizumab

| **Therapeutic** | **Overlap Ratio** | **Adjusted Overlap Ratio** |
| --- | --- | --- |
| Certolizumab | 1.8% | 6.0% |
| Omalizumab | 1.9% | 6.8% |
| Eculizumab | 1.3% | 5.2% |

- 1. Negative control Hu-mAb humanization results

For the full results from the negative control humanization analysis, please see the SI file ‘Negative_Control_Hu-mAb_Results.xlsx’. The precursor and experimentally humanized sequences of the 25 therapeutics used to test Hu-mAb can be found in the SI file ‘Hu-mAb_Results.xlsx’. Abbreviations – FR: framework region, OR: mutation overlap ratio, AOR: adjusted mutation overlap ratio, NC: negative control.

- 1. Analysis of proposed mutations – residue types


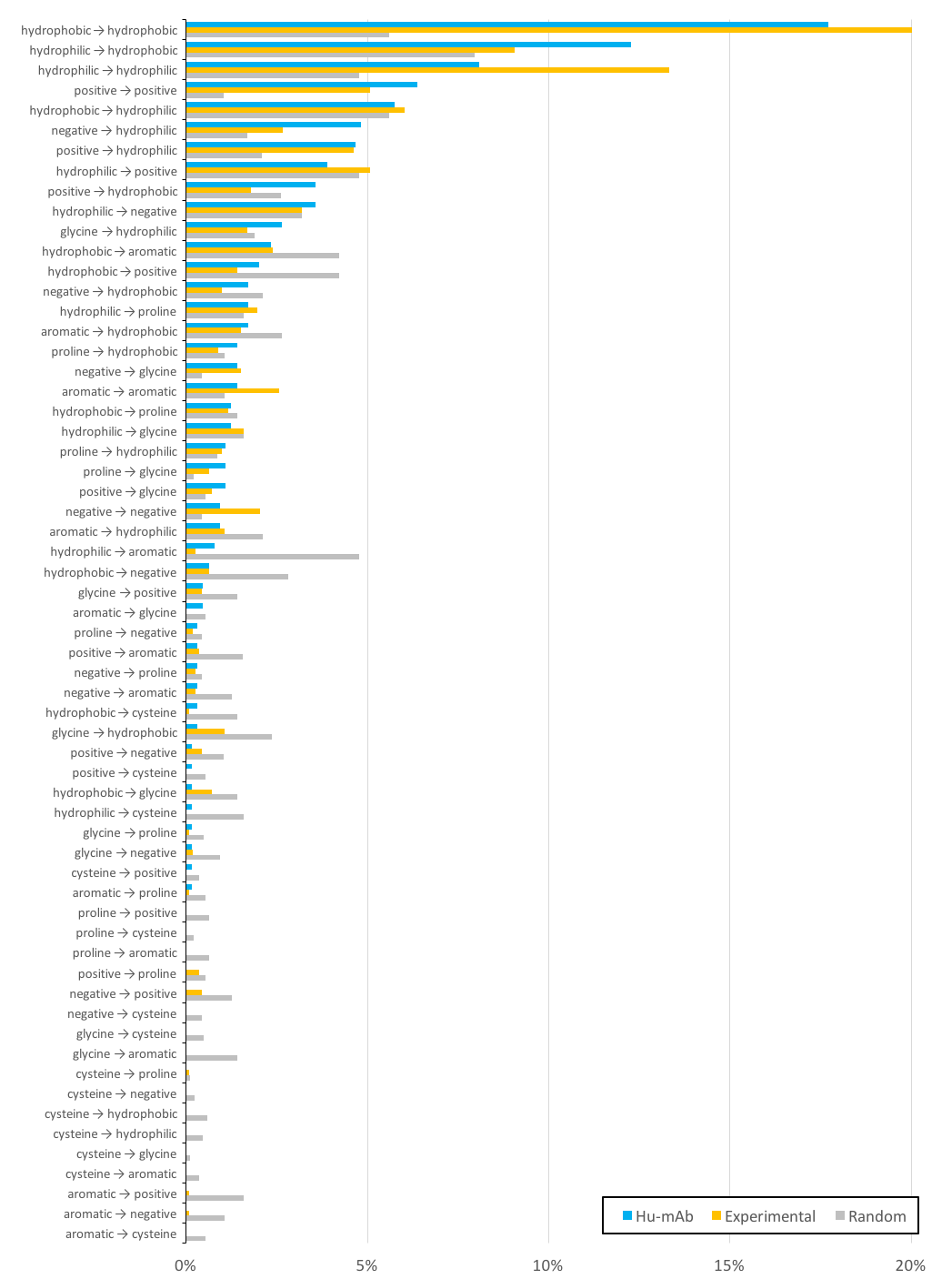


Figure S10. Analysis of all mutations proposed by Hu-mAb and experiment using amino acid groupings given in Table S6. Also shown are the results of performing random mutations on the test sequences (the same number of mutations as proposed by Hu-mAb, repeated 1,000,000 times).

- 1. Analysis of proposed mutations – residue locations

Table S14. Comparison of mutation locations for humanizations performed experimentally and by Hu-mAb.

|  |  | | **VH** | | | | | **VL** | | | | |
| --- | --- | --- | --- | --- | --- | --- | --- | --- | --- | --- | --- | --- |
|  |  | | **Proportion of Mutations** | | **Mutations per Sequence** | | **Overlap Ratio** | **Proportion of Mutations** | | **Mutations per Sequence** | | **Overlap Ratio** |
| **Residues** | | | **Hu-mAb** | **Exp.** | **Hu-mAb** | **Exp.** |  | **Hu-mAb** | **Exp.** | **Hu-mAb** | **Exp.** |  |
| Interface | | Mean | 6.2% | 7.3% | 0.8 | 1.6 | 73.8% | 8.2% | 10.1% | 0.8 | 1.8 | 96.4% |
|  |  | Median | 4.3% | 5.7% | 1.0 | 2.0 | 100.0% | 7.7% | 10.0% | 1.0 | 2.0 | 100.0% |
| Vernier Zone | | Mean | 14.1% | 10.4% | 2.2 | 3.0 | 51.7% | 4.8% | 5.0% | 0.4 | 1.0 | 70.0% |
|  |  | Median | 14.3% | 12.5% | 2 | 3.0 | 58.3% | 0.0% | 4.6% | 0.0 | 1.0 | 100.0% |
| Surface | | Mean | 67.1% | 67.6% | 9.6 | 17.1 | 72.5% | 63.3% | 64.9% | 6.7 | 12.4 | 78.1% |
|  |  | Median | 66.7% | 66.7% | 9.0 | 16.0 | 70.0% | 63.6% | 66.7% | 6.0 | 13.0 | 83.3% |
| Buried | | Mean | 32.8% | 29.8% | 5.1 | 8.1 | 51.5% | 36.7% | 32.6% | 3.9 | 6.3 | 74.3% |
|  |  | Median | 33.3% | 33.3% | 5.0 | 9.0 | 60.0% | 36.4% | 31.3% | 4.0 | 7.0 | 75.0% |

Table S15. Definitions of interface, Vernier zone, surface, and buried residues (IMGT residue numbering).

| **Type** | **VH** | **VL** |
| --- | --- | --- |
| Interface | 44, 47, 48, 52, 101, 107, 109, 114, 116, 117, 120 | 50, 56, 69, 101, 103, 109, 115, 116, 120 |
| Vernier Zone | 2, 52, 53, 54, 76, 78, 80, 82, 87, 118 | 2, 4, 41, 42, 52, 53, 54, 55, 78, 80, 84, 85, 87, 118 |
| Surface | 1, 3, 5, 7, 8, 9, 11, 12, 14, 15, 16, 17, 18, 20, 22, 24, 26, 45, 46, 47, 48, 49, 51, 66, 69, 70, 72, 73, 74, 77, 79, 81, 82, 83, 84, 85, 88, 90, 92, 93, 95, 96, 97, 99, 101, 120, 123, 127, 128 | 1, 3, 5, 7, 8, 9, 10, 11, 12, 14, 15, 16, 17, 18, 20, 22, 24, 26, 45, 46, 47, 48, 51, 66, 67, 69, 70, 72, 73, 74, 77, 79, 80, 81, 82, 83, 84, 85, 86, 88, 90, 92, 93, 95, 96, 97, 101, 120, 123, 127, 128 |
| Buried | 2, 4, 6, 10, 13, 19, 21, 23, 25, 39, 40, 41, 42, 43, 44, 50, 52, 53, 54, 55, 67, 68, 71, 75, 76, 78, 80, 86, 87, 89, 91, 94, 98, 100, 102, 103, 104, 118, 119, 121, 122, 124, 125, 126 | 2, 4, 6, 13, 19, 21, 23, 25, 39, 40, 41, 42, 43, 44, 49, 50, 52, 53, 54, 55, 68, 71, 75, 76, 78, 87, 89, 91, 94, 98, 99, 100, 102, 103, 104, 118, 119, 121, 122, 124, 125, 126 |

Key interface residues were defined according to Raybould et al., 2020.

Vernier zone residues were defined according to Foote and Winter, 1992, converting the numbering scheme from Kabat to IMGT. Residue numbers 28-35 and 105-106 were excluded from the calculation since they are considered part of the CDRs according to the IMGT definition, and hence Hu-mAb would never propose mutations for those residues.

Surface/buried residues were defined using a set of 1129 non-redundant variable domain structures (from Raybould et al., 2020). We calculated the average relative solvent accessibility (RSA) for each position within the structures, and then set a threshold to split the residues between surface exposed and buried. This was defined as 25% of the RSA value, a value which is commonly used for other models (e.g. Wu et al., 2017, Zhang et al., 2017, Bozic et al., 2017).
